# Cigarette smoke extract alters genome‐wide profiles of circular RNAs and mRNAs in primary human small airway epithelial cells

**DOI:** 10.1111/jcmm.14436

**Published:** 2019-05-29

**Authors:** Ni Zeng, Tao Wang, Mei Chen, Zhicheng Yuan, Jiangyue Qin, Yanqiu Wu, Lijuan Gao, Yongchun Shen, Lei Chen, Fuqiang Wen

**Affiliations:** ^1^ Division of Pulmonary Diseases, State Key Laboratory of Biotherapy of China Department of Respiratory and Critical Care Medicine West China Hospital of Sichuan University Chengdu China; ^2^ Department of Respiratory and Critical Care Medicine Chengdu Fifth People’s Hospital Chengdu China

**Keywords:** cigarette smoke, circular RNAs, competing endogenous RNA, small airway epithelial cells

## Abstract

As a novel kind of non‐coding RNA, circular RNAs (circRNAs) were involved in various biological processes. However, the role of circRNAs in the developmental process of chronic obstructive pulmonary disease (COPD) is still unclear. In the present study, by using a cell model of COPD in primary human small airway epithelial cells (HSAECs) treated with or without cigarette smoke extract (CSE), we uncovered 4,379 previously unknown circRNAs in human cells and 903 smoke‐specific circRNAs, with the help of RNA‐sequencing and bioinformatic analysis. Moreover, 3,872 up‐ and 4,425 down‐regulated mRNAs were also identified under CSE stimulation. Furthermore, a putative circRNA‐microRNA‐mRNA network was constructed for in‐depth mechanism exploration, which indicated that differentially expressed circRNAs could influence expression of some key genes that participate in response to pentose phosphate pathway, ATP‐binding cassette (ABC) transporters, glycosaminoglycan biosynthesis pathway and cancer‐related pathways. Our research indicated that cigarette smoke had an influence on the biogenesis of circRNAs and mRNAs. CircRNAs might be involved in the response to CSE in COPD through the circRNA‐mediated ceRNA networks.

## 1 INTRODUCTION

Associated with a high prevalence and related disability and mortality, chronic obstructive pulmonary disease (COPD) has become a worldwide public health challenge. The Global Burden of Disease study reported that an estimated 174.5 million case of COPD occurred worldwide in 2015.^1^, ^2^ A recent Chinese of COPD study indicated that the prevalence of spirometry defined COPD was 8.6% in Chinese adults over 20 years old, accounting for 99.9 million people.^3^ Although cigarette smoking was a major risk factor for COPD, the precise molecular mechanisms remain largely unknown.

Circular RNAs (circRNAs) are a novel group of endogenous non‐coding RNAs, which are originated from linear precursor messenger RNA (pre‐mRNA) and generated by back splicing, with covalently joined 3′‐ and 5′‐ ends.^4^ Unlike liner RNAs, circRNAs are more stable because of their covalently closed loop structures. At first, circRNAs were considered as useless products of abnormal splicing,^5^ recent investigations discovered that circRNAs were widespread and could function as gene expression regulators at multiple levels. For instance, increasing studies revealed that circRNAs could impair miRNA mediated gene silencing by serving as miRNA sponges partially through the competitive endogenous RNA (ceRNA) network.^6^, ^7^ Moreover, the intronic circRNAs, majorly accumulating in the nucleus, could interact with the elongating Pol II complex and thereby influence gene transcription.^8^ Li et al also discovered that intergenic circRNAs could affect Pol II transcription by interacting with small nuclear ribonucleoprotein to promote transcription.^9^

Abundant circRNAs have been discovered in various cells, because of the help of high throughput sequencing technologies and bioinformatic analysis.^8^, ^10^ Subsequent reports revealed that circRNAs may be involved in several cellular and developmental process of some diseases, such as prion diseases, neurological disorders, atherosclerotic vascular disease risk, as well as different malignant tumours.^6^, ^7^, ^11^, ^12^ It is reported that circRNAs could function as potential disease biomarkers in human saliva and as biomarkers for non‐small cell lung cancer and ageing.^13^, ^14^, ^15^ However, the possible involvement of circRNAs in the pathogenesis of COPD remains elusive. In this study, we investigated the expression patterns of circRNAs and mRNAs under cigarette smoking stimulation, a major environmental contributor to COPD, in primary human small airway epithelial cells (HSAECs), to explore the possible underlying molecular regulation mechanism of circRNAs in COPD.

## 2 METHODS AND MATERIALS

### 2.1 Preparation of cigarette smoke extract

We prepared the cigarette smoke extract (CSE) as previously described with some modifications.^16^ Briefly, mainstream smoke derived from five 3R4F Kentucky Research cigarettes was drawn slowly into a 50‐mL syringe and bubbled through 10 mL of Dulbecco's modified Eagle's medium (DMEM) which was pre‐warmed at 37℃. This preparation was next titrated to pH 7.4 and sterilised with a 0.22 μm filter (Millipore, Bedford, MA, USA). The final solution was considered as 100% CSE. Then the 100% CSE was diluted with serum‐free cell culture medium to the required concentrations during use.

### 2.2 Cell culture and stimulation

Primary HSAECs and the BronchiaLife Epithelial Airway Medium Complete Kit including all the growth supplements were purchased from Lifeline Cell Technology. In brief, cells were cultured in basal medium supplemented with 500 µg/mL HSA, 0.6 µM Linoleic Acid, 0.6 µg/mL Lecithin, 6 mM L‐Glutamine LifeFactor, 0.4% Extract P LifeFactor, 1 µM epinephrine, 5 µg/mL transferrin PS, 10 nM T3, 0.1 µg/mL hydrocortisone, 5 ng/mL rh EGF and 5 µg/mL rh insulin, then maintained at 37℃ in a humidified atmosphere containing 5% CO_2_. After serum‐starved overnight, the confluent cells were treated with (Smoke) or without (Control) CSE.

### 2.3 RNA isolation and quantitative real‐time PCR

Total RNA was isolated from HSAECs using EZNA Total RNA kit I (Omega Bio‐tek, USA), as per manufacturer's instructions. cDNAs were synthesised from total RNA using PrimeiScript RT reagent Kit (TaKaRa) following the manufacturer's instructions. Next, the PCR reaction was performed in triplicates with FastStart Essential DNA Green Master (Roche). The relative PCR amplification was determined using the LightCycler^®^ 96 PCR system (Roche Molecular Systems, USA) as following: first step is preincubation: 95°C for 10 minutes for 1 cycle; second step is 3‐step amplification: 95°C for 10 seconds, Tm for 10 seconds and 72°C for 10 seconds for 40 cycles; third step is melting: 95°C for 10 seconds, 65°C for 60 seconds and 97°C for 1 seconds for 1 cycle. Divergent primers were designed and optimised for circRNAs (Table S1). All data were normalised to GAPDH gene expression.

### 2.4 CircRNA‐seq library preparation and RNA sequencing

Total RNA was isolated from HSAECs using EZNA Total RNA kit I (Omega Bio‐tek, USA) per manufacturer's instructions. Then, to eliminate DNA, 50U RNase‐free DNase per μg RNA was added for 15 minutes at 37℃. Furthermore, to purify the circRNAs, the total RNA was treated with 3 U/μg of RNase R (Epicentre) for 20 minutes at 37℃. Subsequently, circRNA was enriched by using acid phenol/chloroform (pH 4.5). After obtaining RNA from HSAECs treated or untreated with CSE for 48 hours (three independent samples for each group), libraries for each condition (Smoke or Control) were generated by NEBNext Ultra directional RNA library Prep kit (NEB) and AMPure XP Beads size selection (Beckman Coulter) as per the manufacturer's protocol. Briefly, a total amount of 5 μg RNA per sample was used as input material for the RNA sample preparations. Fragmentation was carried out using divalent cations under elevated temperature in NEBNext First Strand Synthesis Reaction Buffer. First strand cDNA was synthesised using random hexamer primer and M‐MuLV Reverse Transcriptase (RNase H). Second strand cDNA synthesis was subsequently performed using DNA Polymerase I and RNase H. In the reaction buffer, dNTPs with dTTP were replaced by dUTP. Remaining overhangs were converted into blunt ends via exonuclease/polymerase activities. After adenylation of 3′ ends of DNA fragments, NEBNext Adaptor with hairpin loop structure were ligated to prepare for hybridisation. In order to select cDNA fragments of preferentially 150 ~ 200 bp in length, the library fragments were purified with AMPure XP system (Beckman Coulter, Beverly, USA). Then 3 μL USER Enzyme (NEB, USA) was used with size‐selected, adaptor‐ligated cDNA at 37℃ for 15 minutes followed by 5 minutes at 95℃ before PCR enrichment. Then PCR was performed with Phusion High‐Fidelity DNA polymerase, Universal PCR primers and Index Primer. At last, products were purified (AMPure XP system) and library quality was assessed on the Agilent Bioanalyzer 2100 system. Sequencing was conducted on Illumina Hiseq 4000 by using PE150 mode.

### 2.5 Identification of circRNAs

TopHat version2.1.1 was used to map reads from all samples to the human reference genome (UCSC Genome Browser) as previously described^17^ and CIRCexplore2 software was used to identify candidate circRNAs. Reads supporting candidate circular junctions were then identified together with their strands given the strand‐specific RNA‐Seq data. To increase the confidence of predicted circular junctions, the length of overlap in either junction covered by the corresponding reads was required to be no less than 15 nucleotide (nt). Subsequently, we examined the splice site signals with putative circular junctions. To support the circular junction, if canonical splice site signals (GT, AG) were identified, at least two distinct reads of a putative circular junction in total (summed for three samples) were required to be identified. Otherwise, at least three distinct reads in total were required.

### 2.6 DEGs and GO enrichment analysis

To analyse differentially expressed genes (DEGs), we used DESeq R packages according to the package's protocol. Genes with *P* < 0.05 were considered as differentially expressed. Gene ontology (GO) enrichment analysis of DEGs cover three domains, ie Biological Process (BP), Cellular Component (CC) and Molecular Function (MF), and are based on the hypergeometric test and implemental by the topGO R packages, for a functional analysis, we also carried out the pathway analysis by mapping genes to Kyoto Encyclopedia of Genes and Genomes (KEGG) pathways.

### 2.7 CircRNA‐associated‐ceRNA network prediction

To construct a putative circRNA‐mediated competing endogenous RNA (ceRNA) network, we identified all microRNA (miRNA) targeting circRNA and all miRNA targeting gene based on TargetScan and miRanda. Then, if identical miRNA could bind to both circRNA and gene, the targeted circRNAs were defined as candidate ceRNA of gene, and the circRNA‐miRNA‐mRNA thus represent a candidate ceRNA pairs.^18^ Besides, circRNA and gene in candidate ceRNA pairs are required to have the same direction in DEG analysis, because of a positive correlation in expression of ceRNA pairs.

## 3 RESULTS

### 3.1 Expression pattern of circRNA and mRNA in CSE‐induced stress

We achieved about 55.2 million (from 50.3 to 60.5 million) and 53.8 million (from 46.5 to 58.7 million) mean mapped reads for Control and Smoke, respectively. After further analysis, a total of 10,738 circRNAs were identified from all samples and the length of circRNAs varied from 151 nt to 99934 nt. The number of circRNAs reported in circBase^19^ was 6359 (59.22%), whereas 4,379 (40.78%) circRNA species were newly discovered. Moreover, 4,842 circRNAs were detected in both Smoke and Control, whereas 4,993 and 903 circRNAs were uniquely detected in Control samples and Smoke samples, respectively. The results suggested that CSE remarkably reduced circRNAs accumulation.

**Figure 1:**
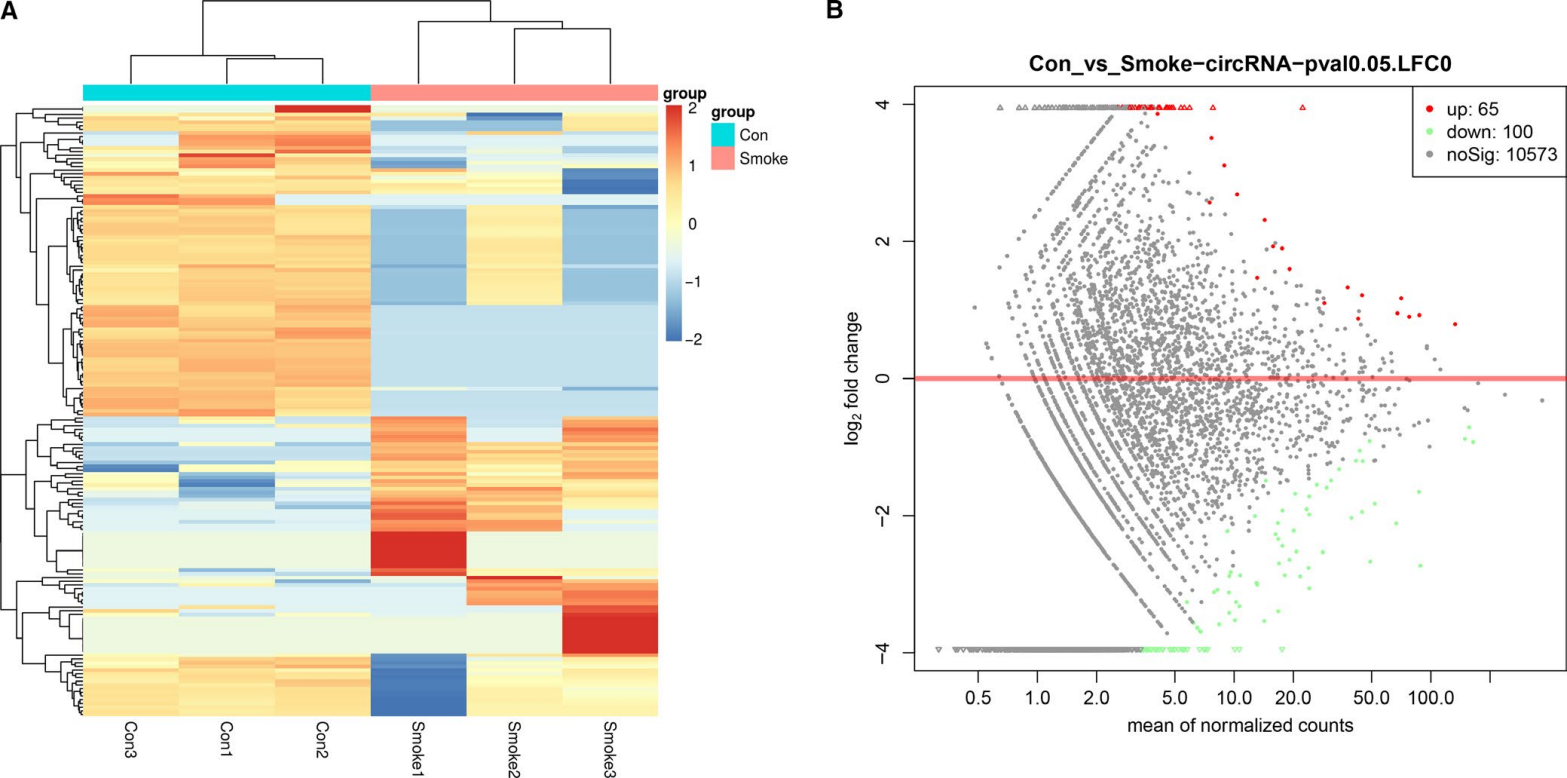
CircRNA expression between Control samples and Smoke samples. A, Heat map of circRNAs in HSAECs treated with or without CSE. Each column represents the expression profiles of a cell sample, and each row corresponds to a circRNA. ‘Red’ indicates higher expression level, ‘blue’ indicates lower expression level. B, MA plots comparing the expression of circRNAs in Control samples and Smoke samples. The red dots represent up‐regulated circRNAs having *P*‐values < 0.05 in the two groups, the green dots represent down‐regulated circRNAs having *P*‐values < 0.05

**Figure 2:**
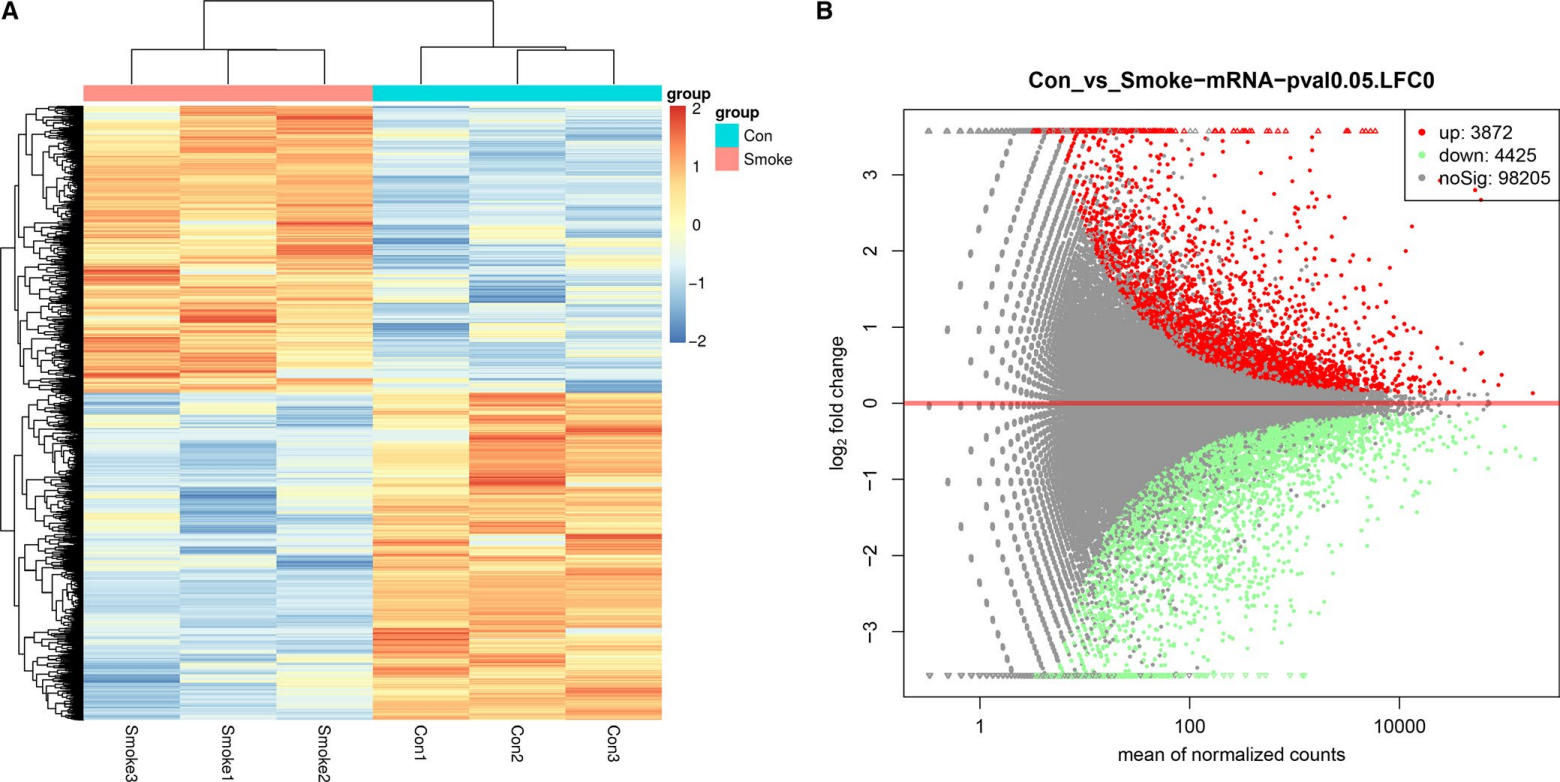
mRNA expression data between Control samples and Smoke samples. A, Heat map of RNA‐seq data was used to assess the significant expression of mRNAs when comparing Smoke samples with Control samples in HSAECs. ‘Red’ and ‘blue’ denotes high and low expression, respectively. B, The MA plots show the number and distribution of differentially expressed mRNAs between the two groups. The red dots represent significantly up‐regulated mRNAs, the green dots represent significantly down‐regulated mRNAs

Among them, there were 65 up‐ and 100 down‐regulated circRNAs (*P* < 0.05) passing MA plots and heat map (Figure 1), in which the top 20 dysregulated circRNAs were summarised in Table 1 based on fold change. In addition, the differential expression of mRNAs was also well categorised into Smoke and Control groups (Figure 2). Three thousand eight hundred and seventy‐two up‐ and 4,425 down‐regulated mRNAs (*P* < 0.05) were identified compared with the control group. The top 20 dysregulated mRNAs were summarised in Table 2.

**Table 1:** Top 10 dysregulated circRNAs

CircRNA	Gene symbol	Log2 fold change(abs)	Chrom	Type
Up regulation				
hsa_circ_0061052	OSBPL2	5.60	chr20	intron
hg38_circ_0011916	n/a^a^	4.65	chr2	Intergenic
hg38_circ_0002169	GCN1	4.56	chr12	exon
hsa_circ_0077520	HACE1	4.52	chr6	intron
hg38_circ_0008840	n/a^a^	4.30	chr1	intergenic
hg38_circ_0002866	RP3‐461F17.3	4.20	chr12	Intron
hg38_circ_0006778	CHMP6	4.16	chr17	Intron
hg38_circ_0006713	AFMID	3.86	chr17	Intron
hg38_circ_0019689	C9orf3	3.51	chr9	Intron
hsa_circ_0008274	n/a^a^	3.11	chr13	intergenic
Down regulation				
hsa_circ_0006892	PI4KB	4.30	chr1	intron
hsa_circ_0006794	FBXO11	3.98	chr2	exon
hsa_circ_0008725	IFNGR2	3.98	chr21	intron
hsa_circ_0004826	UTRN	3.70	chr6	intron
hsa_circ_0008667	ADAMTS6	3.68	chr5	exon
hsa_circ_0085438	TBC1D31	3.63	chr8	intron
hsa_circ_0004422	KANSL1	3.63	chr17	intron
hsa_circ_0002282	FAM53B	3.58	chr10	exon
hsa_circ_0002551	GABPB1	3.56	chr15	intron
hsa_circ_0003060	SUCLG2	3.53	chr3	intron

a n/a: CircRNA produced from intergenic region in the genome.

**Table 2:** Top 10 dysregulated mRNAs in CSE‐induced stress

Gene ID	Gene name	Transcript	Log2 fold change(abs)	*P* value
Up regulation				
ENST00000559279	KIF23	KIF23‐001	7.58	6.09E‐06
ENST00000489960	TPRA1	TPRA1‐005	7.06	5.85E‐05
ENST00000508369	FAM13A	FAM13A‐024	6.82	4.06E‐08
ENST00000437464	ZNF469	ZNF469‐201	6.69	4.75E‐06
ENST00000377532	PER3	PER3‐001	6.50	1.60E‐06
ENST00000439924	IST1	IST1‐011	6.49	3.54E‐08
ENST00000467869	ELL3	ELL3‐003	6.42	0.0079
ENST00000545377	ARHGEF28	ARHGEF28‐202	6.40	0.005131
ENST00000524164	ATP6V1H	ATP6V1H‐009	6.38	0.014951
ENST00000546602	TNS2	TNS2‐008	6.33	9.18E‐05
Down regulation				
ENST00000533129	CAPN1	CAPN1‐028	9.66	0.0175
ENST00000410020	DYSF	DYSF‐007	9.57	0.0003
ENST00000274026	CCNA2	CCNA2‐001	8.99	6.54E‐05
ENST00000593587	HNRNPUL1	HNRNPUL1‐010	8.81	0.0003
ENST00000395006	TMBIM6	TMBIM6‐007	8.66	0.0003
ENST00000610403	RRBP1	RRBP1‐203	8.36	0.005365
ENST00000379059	POLA1	POLA1‐001	8.06	0.02188
ENST00000400319	DIAPH3	DIAPH3‐006	7.58	2.16E‐13
ENST00000589527	LGALS3BP	LGALS3BP‐020	7.48	0.039304
ENST00000340480	HIPK1	HIPK1‐007	7.40	4.60E‐23

### 3.2 Validation of differentially expressed circRNA and time series analysis

**Figure 3:**
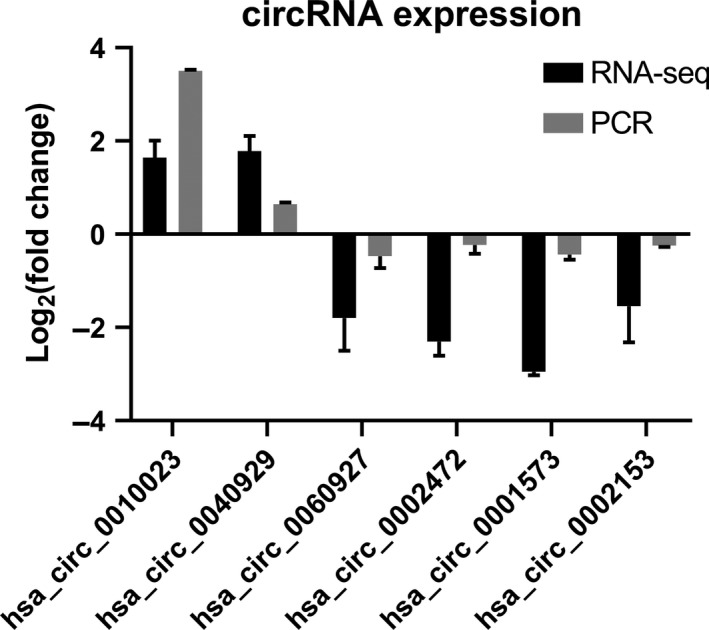
Validation of RNA‐sequencing data by qRT‐PCR. Six differentially expressed circRNAs were validated by qRT‐PCR, including two up‐regulated circRNAs and four down‐regulated circRNAs. The heights of the columns in the chart represent the mean expression value of log2 fold changes (Smoke/Control). All data were normalised to GAPDH gene expression

To verify the RNA sequencing data, we selected six identified circRNAs for the analysis of RT‐qPCR, including two up‐regulated circRNAs and four down‐regulated circRNAs. The results showed that two circRNAs (hsa_circ_0010023 and hsa_circ_0040929) were overexpressed, whereas four circRNAs (hsa_circ_0060927, hsa_circ_0002472, hsa_circ_0002153, hsa_circ_0001573) were underexpressed. The data from RT‐qPCR were consistent with the RNA‐seq data (Figure 3).

Although, circRNA expression was measured at 24, 48 and 72 hours after CSE treatment, the 48 hours measurements gave the strongest enrichment for the majority of selected circRNAs (Figure S1).

### 3.3 Computational analysis of differentially expressed circRNAs and mRNAs

**Figure 4:**
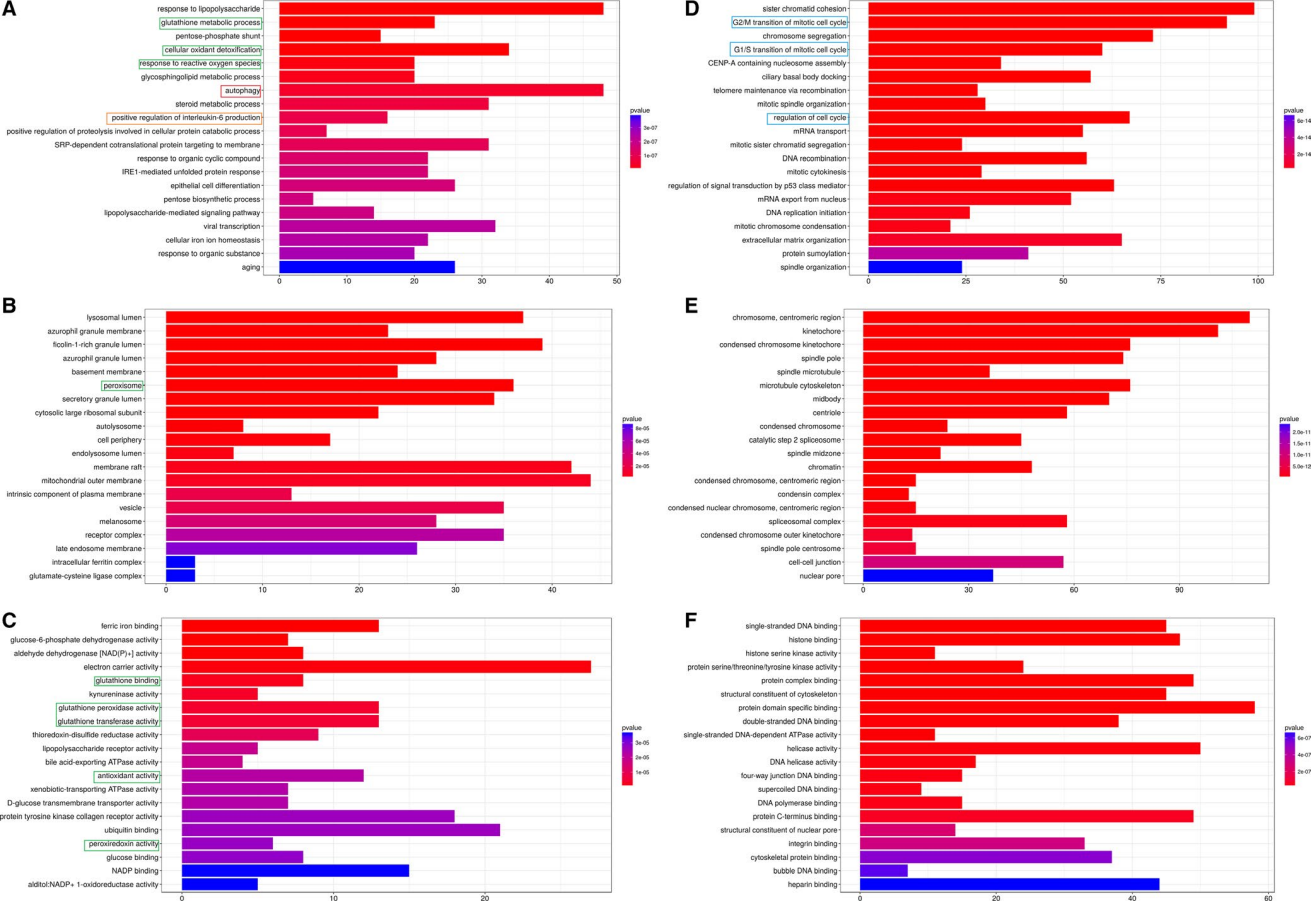
Gene Ontology (GO) enrichment analysis. GO enrichment analysis corresponded to up‐regulated (A, B, C) and down‐regulated (D, E, F) mRNA under the theme of BP (A and D), CC (B and E) and MF (C and F). The graph size represents the number of genes, and the colour represents the *P* value. Green boxes highlight gene clusters involved in response to oxidative stress, Red box highlights gene cluster involved in autophagy, brown box highlights the gene cluster involved in inflammation, blue boxes highlight the gene clusters involved in cell cycles. Twenty gene clusters with most significantly differential expression are shown. BP: Biological Process, CC: Cellular Component, MF: Molecular Function

**Figure 5:**
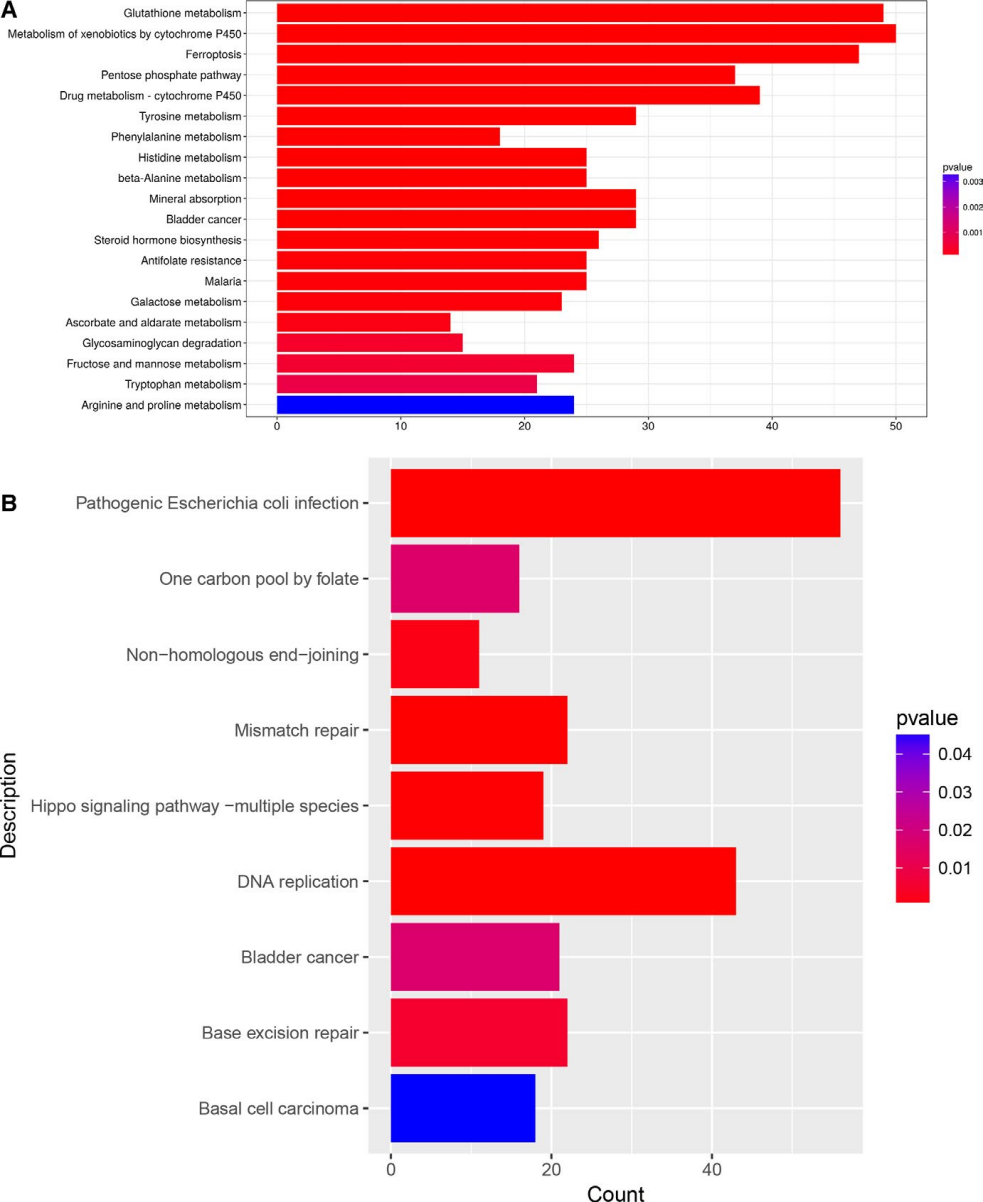
KEGG pathway analysis. KEGG pathways of significantly up‐ (A) and down‐regulated (B) mRNAs. The graph size represents the number of genes, and the colour represents the *P* value

GO enrichment analysis were conducted in terms of Biological Process (BP), Cellular Component (CC) and Molecular Function (MF) to explore the possible functional roles. The top 20 dysregulated GO processes of each subgroup (BP, CC and MF) were analysed according to enriched differentially expressed mRNAs. For increased mRNAs, the top three GO processes included response to lipopolysaccharide, glutathione metabolic, pentose‐phosphate shunt in BP (Figure 4A); lysosomal lumen, azurophil granule membrane, ficolin‐1‐rich granule lumen in CC (Figure 4B) and ferric iron binding, glucose‐6‐phosphate dehydrogenase activity, aldehyde dehydrogenase activity in MF (Figure 4C), following the routine GO classification algorithms (Figure 4). The top 20 GO processes predicted by down‐regulated mRNAs were also shown in Figure 4. The GO analysis revealed that numerous genes were involved in metabolic process and the regulation of biological and cellular process, such as oxidative stress, autophagy, inflammation and cell cycles. Enrichment score was also used to enrich the significant GO terms of significantly dysregulated genes, in which the top three processes were ciliary basal body docking, mRNA transport and response to drug in BP; ficolin‐1‐rich granule lumen, catalytic step 2 spliceosome and spindle midzone in CC; supercoiled DNA binding, RAGE receptor binding and single‐stranded DNA‐dependent ATPase activity in MF (Figure S2). Furthermore, 31 and 9 KEGG pathways were identified in up‐ and down‐regulated mRNAs, respectively (Figure 5).

### 3.4 The prediction of circRNA‐mediated ceRNA networks

**Figure 6:**
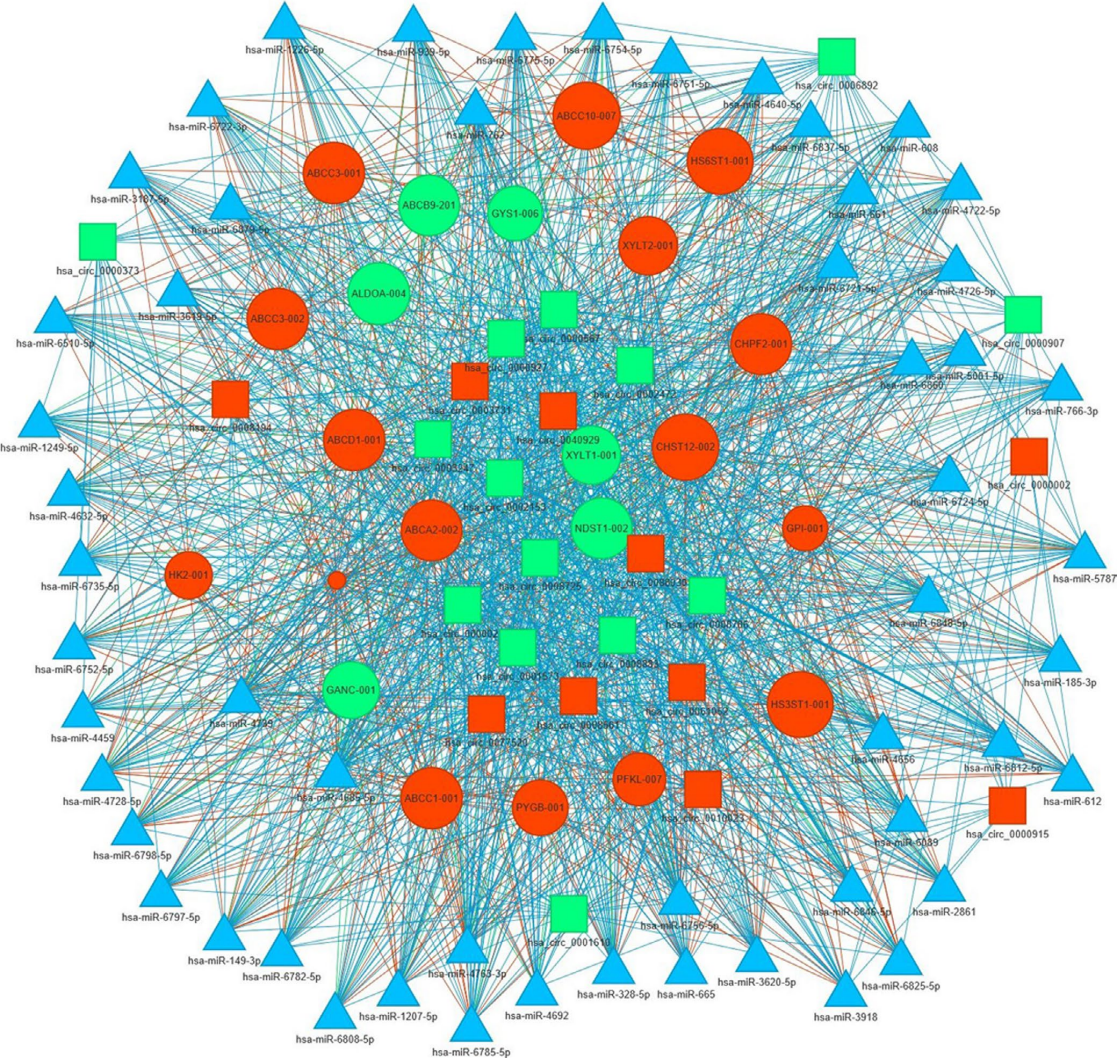
The prediction of circRNA‐associated‐ceRNA networks under CSE stimulation. The ceRNA networks were built on miRNA‐circRNA and miRNA‐gene interactions. Circle, square and triangle indicated gene, circRNA and miRNA, respectively. Red, green and blue indicated up‐regulation, down‐regulation and none detected, respectively

CircRNAs could function as miRNA sponges and inhibit miRNA activity mediated by ceRNA network. In the present study, we predicted the circRNA‐mediated ceRNA networks in HSAECs under CSE stimulation (Figure 6). Firstly, all the candidate ceRNA pairs that cirRNA could target miRNA and miRNA could target mRNA were identified. Considering that the circRNAs and mRNAs should have similar expression patterns response to CSE, both circRNA and mRNA are up‐ or down‐regulated in one ceRNA pair, we next screened these potential ceRNA pairs by DEG analysis. As shown in Figure 6, about 24 circRNAs were connected to expression of 21 genes through several miRNAs. These target genes were found to be associated in multiple pathways including pentose phosphate pathway, ATP‐binding cassette (ABC) transporters, glycosaminoglycan biosynthesis pathway and cancer‐related pathways, as indicated by GO enrichment analysis.

## 4 DISCUSSION

As a newly identified group of widespread endogenous non‐coding RNAs, circRNAs were proved to regulate their parent gene expression to affect disease. The potential importance of circRNAs was reported in many types of diseases. However, there are no reported studies on the functional roles of circRNAs in COPD. Long‐term cigarette smoke exposure is the most important risk factor in the development of COPD. In this study, we explored the expression patterns of circRNAs under smoke stress in primary HSAECs to investigate the possible mechanisms of COPD. Differentially expressed profiles of circRNAs and mRNAs in HSAECs with CSE treatment were observed compared with control groups, which indicates that these dysregulated circRNAs and mRNAs possibly involve in the development of dysfunction of small airway epithelial cells in patient with COPD.

Accumulating evidence proved that circRNAs played an important role in biological development and multiple cellular processes.^6^, ^10^, ^20^ By high throughput sequencing technologies and bioinformatic analysis, many circRNAs were discovered in different human cells. Some circRNAs displayed stress‐specific expression patterns. For instance, 37 circRNAs were differentially expressed in peripheral blood mononuclear cells in active tuberculosis patients.^21^ In human umbilical venous endothelial cells, a significant up‐regulation of circRNAs was identified to respond to hypoxia.^22^ Here we report, for the first time, the circRNA and mRNA transcriptome profiles of the HSAECs under stimulation of CSE. In our study, a total of 10,738 circRNAs were identified in HSAECs. Among them, 4,384 cicRNAs were previously unknown in human cells. Besides, less circRNAs were expressed in Smoke groups than in Control groups, indicating that cigarette smoke reduced the biogenesis of circRNAs remarkably. Further analysis revealed that there were 65 circRNAs significantly up‐regulated and 100 circRNAs significantly down‐regulated in Smoke groups compared with control groups, respectively. In which, hsa_circ_0061052, hg38_circ_0011916 and hg38_circ_0002169 were up‐regulated with top magnitudes, hsa_circ_0006892, hsa_circ_0006794 and hsa_circ_0008725 were down‐regulated with top magnitudes. Our results indicated that the differential expression patterns of circRNAs may be related to their involvement in the process of COPD with smoke inhalation.

Data from mRNA sequencing were also informative. Plenty of studies performed RNA‐seq to explore non‐coding RNA and mRNA expression profiles in COPD patients’ lungs and blood samples. We compared our list of differentially expressed mRNA with those previously reported in COPD lung tissues and blood samples. The mRNAs including IL6, S100A9 and SPP1, which were differentially expressed in our study, were also identified in COPD patients’ lung tissues.^23^ Furthermore, it is reported that FAM13A in the top up‐regulated mRNAs could increase emphysema susceptibility of mice exposing to cigarette smoke by promoting the degradation of β‐catenin,^24^ a protein involved in cigarette smoke‐induced inflammatory cytokine production and airway inflammation in COPD.^16^ A previous study also suggested that FAM13A polymorphisms might be associated with COPD susceptibility in Chinese Han population.^25^ Moreover, previous studies revealed the role of TMBIM6 in apoptosis progression and endoplasmic reticulum stress. It is considered that both apoptosis and endoplasmic reticulum stress play important roles in the development of COPD. All the correlative data provide clues for the underlying mechanism of COPD.

Furthermore, results from GO enrichment analysis and KEGG pathway also help to enrich important mRNAs in CSE‐induced stress in HSAECs. In our GO process, certain gene clusters involved in response to oxidative/nitrosative stress were enriched. Oxidative and nitrosative stress have been suggested as factors in the development of COPD. For example, Seimetz et al reported that inducible nitric oxide synthase (iNOS) contributed to pulmonary hypertension, emphysema in COPD mice model caused by tobacco‐smoke exposure.^26^ In the top GO processes of increased mRNAs, autophagy identified in BP was reported to promote lung epithelial cell death, airway dysfunction in the experimental models of COPD exposed to cigarette smoke in vivo and in vitro.^27^ In addition, as a part of endoplasmic reticulum stress, IRE1‐mediated unfolded protein response was suggested involved in CSE‐induced apoptosis in alveolar type II epithelial cells.^28^ In the GO processes of decreased mRNAs, regulation of signal transduction by p53 class mediator process and extracellular matrix organisation process were identified in BP whereas histone serine kinase activity and RAGE receptor binding were found in MF, which is corresponding to the previous studies.^29^ Concerning KEGG pathway, ATP‐binding cassette (ABC) transporters in increased mRNAs^30^ showed their importance in CSE‐induced stress. All these findings provided novel clues for COPD study.

Studies using different models have shown that miRNAs expression could be affected by direct exposure to cigarette smoke or exposure to CSE in the lungs. For instance, a decreased miR200c was found to regulate an increase in epithelial to mesenchymal transition (EMT) during NF‐κB‐mediated inflammation in human bronchial epithelial cells induced by CSE, and the increased EMT is associated with pathological processes and tissue remodelling.^31^ Another miRNA molecular, miR135b was reported to serve as a counter‐regulatory mechanism by regulating the IL‐1 receptor expression for cigarette smoke‐induced inflammation in vivo.^32^ The possible relationship between miRNAs and the related target genes with COPD has been supported by large numbers of studies using both animal models and human samples, which were recently well summarised.^33^

CircRNAs were reported to serve as miRNA sponges and keep miRNA away from its target genes through ceRNA network,^11^ suggesting that they might also play important roles in the development of COPD associated with smoke inhalation, considering the close linkage between miRNA and COPD. The circRNA‐mediated ceRNA networks were predicted in several types of human cells.^34^, ^35^, ^36^ In the present study, we also constructed a putative circRNA‐mediated ceRNA network in HSAECs under cigarette smoke stimulation. As shown in Figure 6, circRNAs could influence expression of many genes which were required for pentose phosphate pathway, ATP‐binding cassette (ABC) transporters, glycosaminoglycan biosynthesis pathway and other pathways, by interacting with miRNAs. Our results suggested that circRNA‐mediated post‐transcriptional regulations might be involved in the process of COPD through such miRNAs in airway epithelial cells. Although we could not conclude whether these circRNAs are specific responses to CSE exposure, comparison of the circRNAs expression profiles among different stimulus such as CSE and LPS is well worth being performed in the future study.

Some limitations in this study should be acknowledged. First, it is reported that different circRNA detection tools result in different sets of circRNAs, and several algorithms combined could contribute to ideally reliable predictions.^37^ In the present study, only CIRCexplore2 was used to identify candidate circRNAs, and combination of multiple tools may increase the robustness of circRNA detection. Second, this study only identified the circRNAs and mRNAs expression profiles in small airway epithelial cells. Although these findings provide an important research basis for the future investigation of the pathological and molecular mechanisms underlying the regulation of the development of COPD by these specific circRNAs and mRNAs molecules, further verification in animals and patients is required.

## 5 CONCLUSION

In conclusion, a unique set of circRNAs and mRNAs expression patterns were found in primary HSAECs treated with CSE and these dysregulated circRNAs might participate in airway cells response to cigarette smoke‐induced stress by post‐transcriptional regulation of ceRNA networks. Moreover, our results provided new clues for investigating circRNAs functions in COPD.

## COMPETING INTERESTS

All authors declare that they have no competing interests.

## Data Availability

All data used to support the findings of the current study are available from the corresponding authors upon request.

## References

[1] Collaborators GD a I I a P . Global, regional, and national incidence, prevalence, and years lived with disability for 310 diseases and injuries, 1990–2015: a systematic analysis for the Global Burden of Disease Study 2015. Lancet. 2016;388(10053):1545‐1602. 27733282 10.1016/S0140-6736(16)31678-6 PMC5055577

[2] Collaborators G C R D . Global, regional, and national deaths, prevalence, disability‐adjusted life years, and years lived with disability for chronic obstructive pulmonary disease and asthma, 1990–2015: a systematic analysis for the Global Burden of Disease Study 2015. Lancet Respir Med. 2017;5(9):691‐706. 28822787 10.1016/S2213-2600(17)30293-X PMC5573769

[3] Wang C , Xu J , Yang L , et al. Prevalence and risk factors of chronic obstructive pulmonary disease in China (the China Pulmonary Health [CPH] study): a national cross‐sectional study. Lancet. 2018;391(10131):1706‐1717. 29650248 10.1016/S0140-6736(18)30841-9

[4] Chen LL . The biogenesis and emerging roles of circular RNAs. Nat Rev Mol Cell Biol. 2016;17(4):205‐211. 26908011 10.1038/nrm.2015.32

[5] Cocquerelle C , Mascrez B , Hetuin D , Bailleul B . Mis‐splicing yields circular RNA molecules. FASEB J. 1993;7(1):155‐160. 7678559 10.1096/fasebj.7.1.7678559

[6] Memczak S , Jens M , Elefsinioti A , et al. Circular RNAs are a large class of animal RNAs with regulatory potency. Nature. 2013;495(7441):333‐338. 23446348 10.1038/nature11928

[7] Hansen TB , Jensen TI , Clausen BH , et al. Natural RNA circles function as efficient microRNA sponges. Nature. 2013;495(7441):384‐388. 23446346 10.1038/nature11993

[8] Zhang Y , Zhang X‐O , Chen T , et al. Circular intronic long noncoding RNAs. Mol Cell. 2013;51(6):792‐806. 24035497 10.1016/j.molcel.2013.08.017

[9] Li Z , Huang C , Bao C , et al. Exon‐intron circular RNAs regulate transcription in the nucleus. Nat Struct Mol Biol. 2015;22(3):256‐264. 25664725 10.1038/nsmb.2959

[10] Salzman J , Gawad C , Wang PL , Lacayo N , Brown PO . Circular RNAs are the predominant transcript isoform from hundreds of human genes in diverse cell types. PLoS ONE. 2012;7(2):e30733. 22319583 10.1371/journal.pone.0030733 PMC3270023

[11] Hansen TB , Kjems J , Damgaard CK . Circular RNA and miR‐7 in cancer. Cancer Res. 2013;73(18):5609‐5612. 24014594 10.1158/0008-5472.CAN-13-1568

[12] Burd CE , Jeck WR , Liu Y , Sanoff HK , Wang Z , Sharpless NE . Expression of linear and novel circular forms of an INK4/ARF‐associated non‐coding RNA correlates with atherosclerosis risk. PLoS Genet. 2010;6(12):e1001233. 21151960 10.1371/journal.pgen.1001233 PMC2996334

[13] Westholm J , Miura P , Olson S , et al. Genome‐wide analysis of drosophila circular RNAs reveals their structural and sequence properties and age‐dependent neural accumulation. Cell Rep. 2014;9(5):1966‐1980. 25544350 10.1016/j.celrep.2014.10.062 PMC4279448

[14] Bahn JH , Zhang Q , Li F , et al. The landscape of microRNA, Piwi‐interacting RNA, and circular RNA in human saliva. Clin Chem. 2015;61(1):221‐230. 25376581 10.1373/clinchem.2014.230433 PMC4332885

[15] Hang D , Zhou J , Qin NA , et al. A novel plasma circular RNA circFARSA is a potential biomarker for non‐small cell lung cancer. Cancer Med. 2018;7(6):2783‐2791. 29722168 10.1002/cam4.1514 PMC6010816

[16] Guo L , Wang T , Wu Y , et al. WNT/β‐catenin signaling regulates cigarette smoke‐induced airway inflammation via the PPARδ/p38 pathway. Lab Invest. 2015;96:218‐229. 26322419 10.1038/labinvest.2015.101

[17] Pan T , Sun X , Liu Y , et al. Heat stress alters genome‐wide profiles of circular RNAs in Arabidopsis. Plant Mol Biol. 2018;96(3):217‐229. 29177640 10.1007/s11103-017-0684-7

[18] Li J‐H , Liu S , Zhou H , Qu L‐H , Yang J‐H . starBase v2. 0: decoding miRNA‐ceRNA, miRNA‐ncRNA and protein‐RNA interaction networks from large‐scale CLIP‐Seq data. Nucleic Acids Res. 2014;42(D1):D92–D97. 24297251 10.1093/nar/gkt1248 PMC3964941

[19] Glazar P , Papavasileiou P , Rajewsky N . circBase: a database for circular RNAs. RNA. 2014;20(11):1666–1670. 25234927 10.1261/rna.043687.113 PMC4201819

[20] Zhang X‐O , Wang H‐B , Zhang Y , Lu X , Chen L‐L , Yang LI . Complementary sequence‐mediated exon circularization. Cell. 2014;159(1):134–147. 25242744 10.1016/j.cell.2014.09.001

[21] Huang Z‐K , Yao F‐Y , Xu J‐Q , et al. Microarray expression profile of circular RNAs in peripheral blood mononuclear cells from active tuberculosis patients. Cell Physiol Biochem. 2018;45(3):1230–1240. 29448254 10.1159/000487454

[22] Boeckel J‐N , Jaé N , Heumüller AW , et al. Identification and characterization of hypoxia‐regulated endothelial circular RNA. Circ Res. 2015;117(10):884–890. 26377962 10.1161/CIRCRESAHA.115.306319

[23] Ezzie ME , Crawford M , Cho J‐H , et al. Gene expression networks in COPD: microRNA and mRNA regulation. Thorax. 2012;67(2):122–131. 21940491 10.1136/thoraxjnl-2011-200089

[24] Jiang Z , Lao T , Qiu W , et al. A chronic obstructive pulmonary disease susceptibility gene, FAM13A, regulates protein stability of beta‐catenin. Am J Respir Crit Care Med. 2016;194(2):185–197. 26862784 10.1164/rccm.201505-0999OC PMC5003213

[25] Wang BO , Liang B , Yang J , et al. Association of FAM13A polymorphisms with COPD and COPD‐related phenotypes in Han Chinese. Clin Biochem. 2013;46(16–17):1683–1688. 23891779 10.1016/j.clinbiochem.2013.07.013

[26] Seimetz M , Parajuli N , Pichl A , et al. Inducible NOS inhibition reverses tobacco‐smoke‐induced emphysema and pulmonary hypertension in mice. Cell. 2011;147(2):293–305. 22000010 10.1016/j.cell.2011.08.035

[27] Ryter SW , Choi AM . Autophagy in lung disease pathogenesis and therapeutics. Redox Biol. 2015;4:215–225. 25617802 10.1016/j.redox.2014.12.010 PMC4803789

[28] Tan S‐X , Jiang D‐X , Hu R‐C , et al. Endoplasmic reticulum stress induces HRD1 to protect Alveolar Type II epithelial cells from apoptosis induced by cigarette smoke extract. Cell Physiol Biochem. 2017;43(4):1337–1345. 28992619 10.1159/000481845

[29] Chen M , Wang T , Shen Y , et al. Knockout of RAGE ameliorates mainstream cigarette smoke‐induced airway inflammation in mice. Int Immunopharmacol. 2017;50:230–235. 28704797 10.1016/j.intimp.2017.06.018

[30] van der Deen M , de Vries E , Timens W , Scheper RJ , Timmer‐Bosscha H , Postma DS . ATP‐binding cassette (ABC) transporters in normal and pathological lung. Respir Res. 2005;6:59. 15967026 10.1186/1465-9921-6-59 PMC1200430

[31] Zhao Y , Xu Y , Li Y , et al. NF‐kappaB‐mediated inflammation leading to EMT via miR‐200c is involved in cell transformation induced by cigarette smoke extract. Toxicol Sci. 2013;135(2):265–276. 23824089 10.1093/toxsci/kft150

[32] Halappanavar S , Nikota J , Wu D , Williams A , Yauk CL , Stampfli M . IL‐1 receptor regulates microRNA‐135b expression in a negative feedback mechanism during cigarette smoke‐induced inflammation. J Immunol. 2013;190(7):3679–3686. 23440414 10.4049/jimmunol.1202456 PMC3607400

[33] Osei ET , Florez‐Sampedro L , Timens W , Postma DS , Heijink IH , Brandsma C‐A . Unravelling the complexity of COPD by microRNAs: it's a small world after all. Eur Respir J. 2015;46(3):807‐818. 26250493 10.1183/13993003.02139-2014

[34] Ghosal S , Das S , Sen R , Chakrabarti J . HumanViCe: host ceRNA network in virus infected cells in human. Front Genet. 2014;5:249. 25120561 10.3389/fgene.2014.00249 PMC4114262

[35] Xu H , Guo S , Li W , Yu P . The circular RNA Cdr1as, via miR‐7 and its targets, regulates insulin transcription and secretion in islet cells. Sci Rep. 2015;5:12453. 26211738 10.1038/srep12453 PMC4515639

[36] Su H , Lin F , Deng X , et al. Profiling and bioinformatics analyses reveal differential circular RNA expression in radioresistant esophageal cancer cells. J Transl Med. 2016;14(1):225. 27465405 10.1186/s12967-016-0977-7 PMC4964270

[37] Hansen TB , Veno MT , Damgaard CK , Kjems J . Comparison of circular RNA prediction tools. Nucleic Acids Res. 2016;44(6):e58. 26657634 10.1093/nar/gkv1458 PMC4824091

