# Supplementary material for: Cigarette smoke extract alters genome‐wide profiles of circular RNAs and mRNAs in primary human small airway epithelial cells
Source: J Cell Mol Med. 2019 May 29;23(8):5532–41. doi: 10.1111/jcmm.14436 (PMC6653042; doi:10.1111/jcmm.14436)
Supplement: Supplementary file 1 [file JCMM-23-5532-s001.docx]

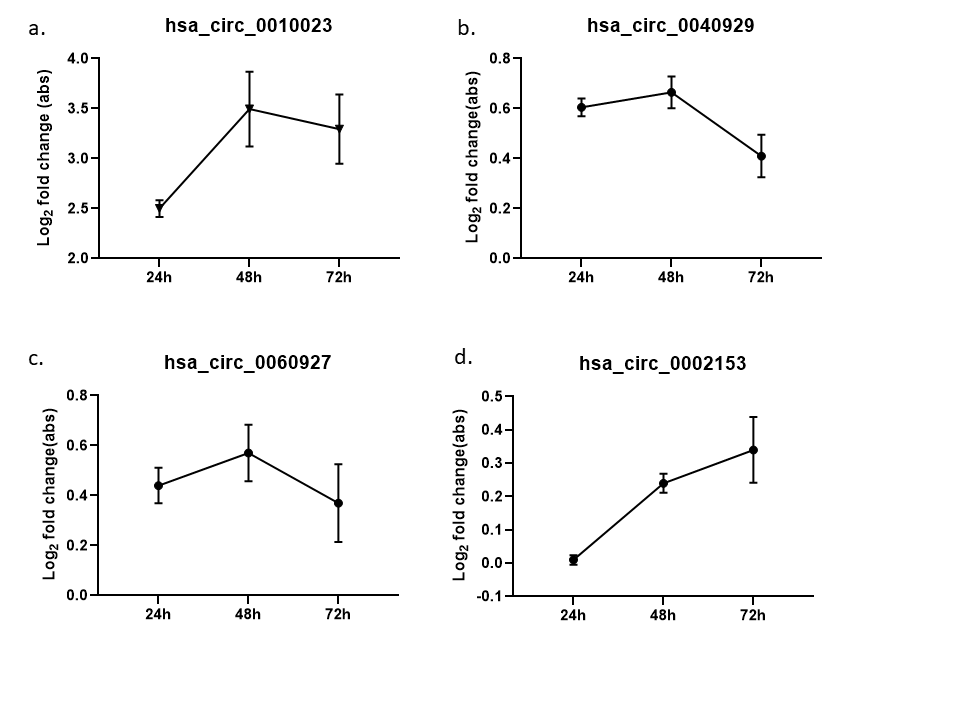


**Figure S1. Time series analysis of circRNA expression levels.**

*Four circRNAs were measured at 24h, 48h and 72h after CSE exposure by qRT-PCR, including 2 upregulated circRNAs (a and b) and 2 downregulated circRNAs (c and d). The heights of the columns in the chart represent the mean absolute expression value of log2 fold changes (Smoke/Control). All data were normalized to GAPDH gene expression.*


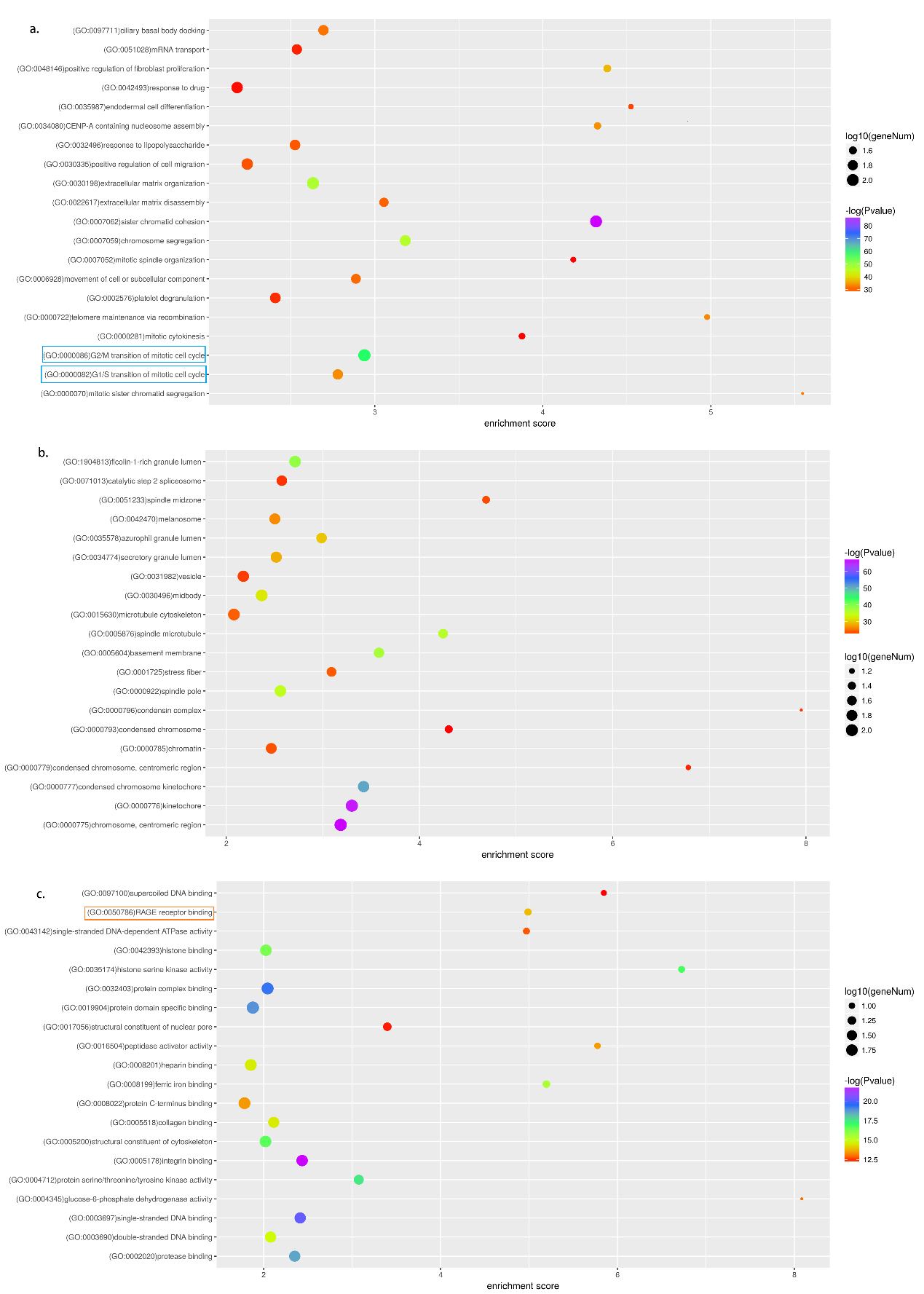


**Figure S2.** ***Go analysis of differential expression mRNAs according to the values in enrichment score***

*Go analysis of differential expression mRNAs according to the values in enrichment score under the theme of BP (a), CC (b) and MF (c). The graph size represents the number of genes, and the color represents the p value. Brown box highlights the gene cluster involved in inflammation, blue boxes highlight the gene clusters involved in cell cycles. 20 gene clusters with most significantly differential expression are shown.*
