# Supplementary material for: Cigarette smoke extract alters genome‐wide profiles of circular RNAs and mRNAs in primary human small airway epithelial cells
Source: J Cell Mol Med. 2019 May 29;23(8):5532–41. doi: 10.1111/jcmm.14436 (PMC6653042; doi:10.1111/jcmm.14436)
Supplement: Supplementary file 2 [file JCMM-23-5532-s002.docx]

**Table S1. The list of primers used in the study**

| **Name** | **Primer sequence (5’-3’)** |
| --- | --- |
| GAPDH (Human) | GGTGGTCTCCTCTGACTTCAACA |
|  | TCTCTTCCTCTTGTGCTCTTGCT |
| hsa_circ_0010023 | TACTGCACATCCAAAGCGTC |
|  | CGGCATTGTTCACCAGGATG |
| hsa_circ_0040929 | AACCAAGACCCCAAAACTGG |
|  | CATCATCAACCGTCTGCTGT |
| hsa_circ_0060927 | ATCCAGGCCACAGACAATGA |
|  | CAGTCTTCCCCTTCCCTGAG |
| hsa_circ_0002472 | GCCTGTCCCCATCGATGATA |
|  | GTTGGCCCTCACATGATTCTT |
| hsa_circ_0002153 | AACCTGGTTAAGCGCAACAG |
|  | CTGGGCTTTCACCTCAACCT |
| hsa_circ_0001573 | TGGCCAGTCATTTACCACCA |
|  | TCAAGCTGCCCTCCTTATTG |
